# Testing of four-sample pools offers resource optimization without compromising diagnostic performance of real time reverse transcriptase-PCR assay for COVID-19

**DOI:** 10.1371/journal.pone.0251891

**Published:** 2021-05-18

**Authors:** Anirudh K. Singh, Ram Kumar Nema, Ankur Joshi, Prem Shankar, Sudheer Gupta, Ashvini Kumar Yadav, Shashwati Nema, Bijina J. Mathew, Arti Shrivas, Chitra Patankar, Arun Raghuwanshi, Ritu Pandey, Ranu Tripathi, Kudsia Ansari, Kuldeep Singh, Jogender Yadav, Debasis Biswas, Sarman Singh

**Affiliations:** 1 Department of Microbiology, All India Institute of Medical Sciences, Bhopal, Madhya Pradesh, India; 2 Regional Virology Laboratory, All India Institute of Medical Sciences, Bhopal, Madhya Pradesh, India; 3 Department of Community and Family Medicine, All India Institute of Medical Sciences, Bhopal, Madhya Pradesh, India; 4 Department of Biochemistry, All India Institute of Medical Sciences, Bhopal, Madhya Pradesh, India; 5 Department of Pediatrics, All India Institute of Medical Sciences, Bhopal, Madhya Pradesh, India; Universidad Nacional de la Plata, ARGENTINA

## Abstract

Quick identification and isolation of SARS-CoV-2 infected individuals is central to managing the COVID-19 pandemic. Real time reverse transcriptase PCR (rRT-PCR) is the gold standard for COVID-19 diagnosis. However, this resource-intensive and relatively lengthy technique is not ideally suited for mass testing. While pooled testing offers substantial savings in cost and time, the size of the optimum pool that offers complete concordance with results of individualized testing remains elusive. To determine the optimum pool size, we first evaluated the utility of pool testing using simulated 5-sample pools with varying proportions of positive and negative samples. We observed that 5-sample pool testing resulted in false negativity rate of 5% when the pools contained one positive sample. We then examined the diagnostic performance of 4-sample pools in the operational setting of a diagnostic laboratory using 500 consecutive samples in 125 pools. With background prevalence of 2.4%, this 4-sample pool testing showed 100% concordance with individualized testing and resulted in 66% and 59% reduction in resource and turnaround time, respectively. Since the negative predictive value of a diagnostic test varies inversely with prevalence, we re-tested the 4-sample pooling strategy using a fresh batch of 500 samples in 125 pools when the prevalence rose to 12.7% and recorded 100% concordance and reduction in cost and turnaround time by 36% and 30%, respectively. These observations led us to conclude that 4-sample pool testing offers the optimal blend of resource optimization and diagnostic performance across difference disease prevalence settings.

## Introduction

Early identification and isolation of infected individuals is key to containing the COVID-19 pandemic [1]. Real time reverse transcriptase polymerase chain reaction (rRT-PCR) remains the gold standard of COVID-19 diagnostics [2]. While the technique is specific, it is too technically demanding to be widely applicable in resource-constrained settings. Paradoxically, the need to ramp up diagnostic capacity is more acute in such settings owing to the higher burden of cases. Furthermore, the second wave of COVID-19 infection experienced by several countries is a testimony that the threat of COVID-19 is far from over [3, 4]. This warrants development of strategies which diagnostic laboratories across the globe can adopt to promptly address the need for increased testing requirement. Although advent of antigen and antibody-based tests can fulfill the requirements of rapid testing, their overall applicability is limited by sub-optimal diagnostic sensitivity [5–7].

Testing of pooled samples by rRT-PCR can offer a potential solution provided it demonstrates optimum diagnostic performance. Pooling has already been used for infectious diseases such as influenza and acquired immunodeficiency syndrome [8, 9]. In fact, in an attempt to facilitate more testing with quicker turn-around time, Federal Drug Administration, USA, recommended pool testing of asymptomatic individuals as early as June 16, 2020 [10]. Several studies have also evaluated the utility of pooled testing for COVID-19 diagnosis. While all of these studies found that pooling reduces the cost and turnaround time, observations regarding the diagnostic sensitivity of pooled testing varied [11–17]. This variation is probably due to differences in background prevalence of the disease and pool size. Thus, deciding the appropriate pool size has always been a contentious issue as it involves balancing between optimal use of resources and maintaining the diagnostic sensitivity and specificity of the assay. In our previous study we observed that using a pool size of five samples saved two-thirds of the cost and time, at the cost of 4% false negative results [15].

Considering the public health ramification of failing to detect infected individuals, we set out to determine the pool size that enables optimization of the resources without compromising the diagnostic performance of rRT-PCR.

## Materials and methods

### Sample collection

The study was approved by the Institutional Human Ethics Committee of All India Institute of Medical Sciences, Bhopal with the waiver of consent as per the “National Ethical Guidelines for Biomedical and Health Research” from Indian Council of Medical Research, a government body which makes and enforces policies of medical research in India. As per the guidelines, a waiver of consent may be granted by institutional ethics committee if the research is done on anonymized biological samples and/or the primary purpose of the research is refinement and improvement of the public health programs. As our study met both these criteria it the waiver of consent was granted. The study was conducted in 2 phases; (a) pooled testing of simulated 5-sample pools, and (b) comparative evaluation of individualized and 4-sample pooled testing strategies. Nasopharyngeal and/or oropharyngeal swabs samples from COVID-19 suspects were collected by trained healthcare workers in Viral Transport Medium (VTM) and transported at 2–8°C to the Regional Virology Laboratory, All India Institute of Medical Sciences, Bhopal. All samples used in this study were anonymized before being accessed by the research team. For the first phase of the study archived samples were used as described below, while the second phase was undertaken on freshly collected samples.

### Simulated pooling of retrospective samples

In order to find out the relative performance of 5-sample pools comprising of varying proportions of positive and negative samples, we randomly chose 50 anonymized positive and 415 negative samples from our departmental repository collected between first week of April and second week of May 2020. These samples were accessed on May 20–27, 2020 for the experiments. We used a web-based random number generator to create simulated pools of different constitutions with positive and negative samples as depicted in Table 1 by mixing 200 μl of each sample in a microfuge tube.

**Table 1:** Concordance between individualized and simulated pooling strategies, using 5-sample pools.

Composition of pools	No. of pools	Mean (SD) Ct value of positive samples on individualized testing	Median (IQR) Ct value of positive samples on individualized testing	Mean (SD) Ct value of positive pool(s)	Median (IQR) Ct value of positive pool(s)	Concordance (%)
5 Negatives	50	NA	NA	37.14	37.14	49/50 (98%)
4 Negatives+ 1 Positive	20	30.04	31.47	32.06	33.99	19/20 (95%)
		(5.68)	(24.4–34.73)	(5.81)	(26.42–35.40)	
3 Negatives+ 2 Positives	20	30.21	31.91	27.27	25.80	20/20 (100%)
		(6.57)	(23.63)	(5.56)	(25.09–34.76)	
2 Negatives + 3 Positives	10	31.49	32.86	29.12	27.89	10/10 (100%)
		(5.75)	(25.60–36.67)	(5.01)	(25.96–32.87)	
1 Negative + 4 Positives	5	30.77	31.81	25.50	25.18	5/5
		(6.27)	(24.49–36.39)	(2.47)	(24.84–27.58)	(100%)
5 Positives	5	30.76	31.85	23.81	24.23	5/5
		(6.63)	(23.64–36.43)	(1.94)	(23.03–25.31)	(100%)

### Prospective consecutive pooling

For this part of the study, aliquots of 1000 consecutive samples were anonymized and processed parallelly for individualized and pooled testing in a blinded manner. To evaluate the sensitivity of pooled analysis in two different prevalence settings, 500 samples were collected in June 2020 (scenario 1) and another 500 in August 2020 (scenario 2) from the same geographical region. A total of 250 pools of 4 anonymized consecutive samples each (125 each in June and August 2020) were thus created by mixing 200 μl of each sample and tested for SARS-CoV-2 along with individual samples as described earlier [15].

### rRT-PCR for COVID-19 diagnosis

RNA from the simulated pools and the samples of scenario 1 were extracted using MGI kit (BGI Biotechnology, Wuhan, China) as per the manufacturer’s protocol and subjected to COVID-19 diagnosis using Lab Gun® fluorescent rRT-PCR diagnostic kit (Lab Genomics, Suwon-si, Republic of Korea). RNA from samples collected in scenario 2 were extracted using Hi-Media Viral RNA extraction kit (Hi-Media) and rRT-PCR assays were performed using Meril Diagnostic (Vapi, India) and 3B BlackBio Biotech (Bhopal, India) as per the kit protocol.

### Calculation of reduction in cost and turnaround time for COVID-19 rRT-PCR test

For calculating the expenditure of rRT-PCR test, cost of nucleic acid extraction kits; RT-PCR kits and other laboratory consumables were taken in the account. In a single 96 well PCR plate 93 samples and three controls were run and total time to taken for one run was 6.5 h which included time for sample aliquoting, RNA extraction and performing the PCR.

### Statistics

The quantification of agreement and bias between the individual Ct value and pool Ct value was determined by Bland-Altman (BA) plot. X-axis of BA plot represented mean Ct value of individual sample and pool while Y-axis represented difference in individual and pool Ct value. We estimated the agreement interval at 95% confidence interval (CI) and each x-y paired observation was colored as per the number of rRT-PCR positive sample(s) in the pool. The ‘BlandAltmanLeh’ and ‘ggplot2’ package with base R software was used to draw the plot.

In the next step the Kendell’s tau coefficient between individual Ct value and pooled Ct value was determined. The reason for choosing Kendell’s tau over other correlation measure (Pearson or Spearman measure) was influenced by the construct of the study where the estimated probability of agreeability (concordance) between the paired individual and pooled Ct value is a major concern. Kendell’s tau, being a distribution-free (independent) non-parametric quantile base measure, seems more robust in this scenario. We used ‘ggpubr’ in R for this purpose. In order to visualize the effect of number of RT-PCR positive samples in 4- and 5-sample pools on Ct value separately, we created the best fit line for nested subgroup as per number of positive samples in both 4-sample and 5-sample pools.

## Results

### Five-sample pool testing strategy for COVID-19 compromises on clinical sensitivity

In our previous study we tested the diagnostic performance of 5-sample pooling strategy for COVID-19 and observed it to be a function of the proportion of positive samples in the pool and the Ct value of the positive sample. Pools containing a single positive sample with Ct value near the cut-off were more likely to give false negative results [15]. While pools with more than one positive sample gave 100% concordance with individualized testing, the study suffered from the limitation of including relatively small number of pools with ≥ 1 positive sample. In order to evaluate the performance of 5-sample pool strategy of COVID-19 diagnosis with varying pool compositions, we adopted a simulated 5-sample pooling strategy with known negative and positive samples from our departmental repository. Of the 110 pools created, 50 pools comprised of all negative samples and 60 had varying proportions of negative and positive samples (Table 1). While all the pools with more than one positive sample showed 100% concordance with the individualized sample testing strategy, the concordance dropped in pools with no positive and one positive sample showing 98% (49/50) and 95% (19/20) concordance, respectively. Expectedly, the Ct values of pooled samples were found to be lower than that of individual positive samples in pools with multiple positive samples (Table 1).

### Four-sample pool testing is comparable to individualized testing of COVID-19

The compromised sensitivity observed with 5-sample pool testing in our previous study [15] and in this study, was limited to the pools containing a single positive sample of high Ct value. We surmised that the false negative results could be due to the dilution of the positive sample in the pool of five. Accordingly, to reduce the dilutional effect, we examined a pool size of 4 samples and tested 500 consecutive samples individually as well as in pool of four consecutive samples from Central India during the month of June 2020 when the background prevalence of COVID-19 in the area was 2.7% (Scenario-1). Out of 125 pools, 115 contained no positive sample, eight pools had one positive sample and two pools had two positive samples. We observed 100% concordance between individualized and pooled sample testing strategies (Table 2).

**Table 2:** Concordance between individualized and consecutive pooling strategies of 4-sample pools in 2 different prevalence settings.

	Pools	Composition of pools	No. of pools	Mean (SD) Ct value of Positive samples	Median (IQR) Ct value of Individuals	Mean (SD) Ct values of pools	Median (IQR) Ct value of pools	Concordance (%)
**Scenario 1** (Background community prevalence: 2.7%)	125	4 Negatives	115	NA	NA	NA	NA	115/115 (100%)
		3 Negatives + 1 Positive	8	31.64 (4.64)	31.64 (29.32–32.95)	34.44 (4.16)	34.44 (31.82–36.66)	8/8 (100%)
		2 Negatives + 2 Positives	2	38.15 (3.39)	38.15 (37.91–40)	37.20 (1.37)	37.20 (36.72–37.69)	2/2 (100%)
**Scenario 2** (Background community prevalence: 12.4%)	125	4 Negatives	76	NA	NA	NA	NA	76/76 (100%)
		3 Negatives+ 1 Positive	24	25.71 (4.71)	25.73 (23.39–28.20)	27.5 (4.43)	27.19 (25.15–30.91)	24/24 (100%)
		2 Negatives+ 2 Positives	16	25.46 (5.48)	24.70 (21.52–29.69)	25.68 (4.15)	26.09 (22.54–27.76)	15/15(100%)
		1 Negative + 3 Positives	5	26.49 (5.29)	25.65 (23.14–31.96)	27.36 (3.95)	27.69 (25.42–30.52)	5/5 (100%)
		4 Positives	4	23.88 (8.23)	22.59 (16.16–32.27)	19.45 (4.18)	19.37 (16.34–22.48)	4/4 (100)

NA: Not Applicable

The negative predictive value of a diagnostic test is inversely related to the background prevalence of the disease. To evaluate the utility of 4-sample pool testing in a different background prevalence setting viz. 12.4% in the same geographical area, we further tested 500 consecutive samples individually and in the pools of 4 in the month of August 2020 (Scenario 2). We observed 100% concordance of results between 4-samples pool and individualized testing. A total of 86 positive samples were assorted in 49 positive pools, among which 25 contained one positive sample, 15 contained two positive samples, five contained 3 positive samples and four had all positive samples (Table 2).

**Fig 1:**
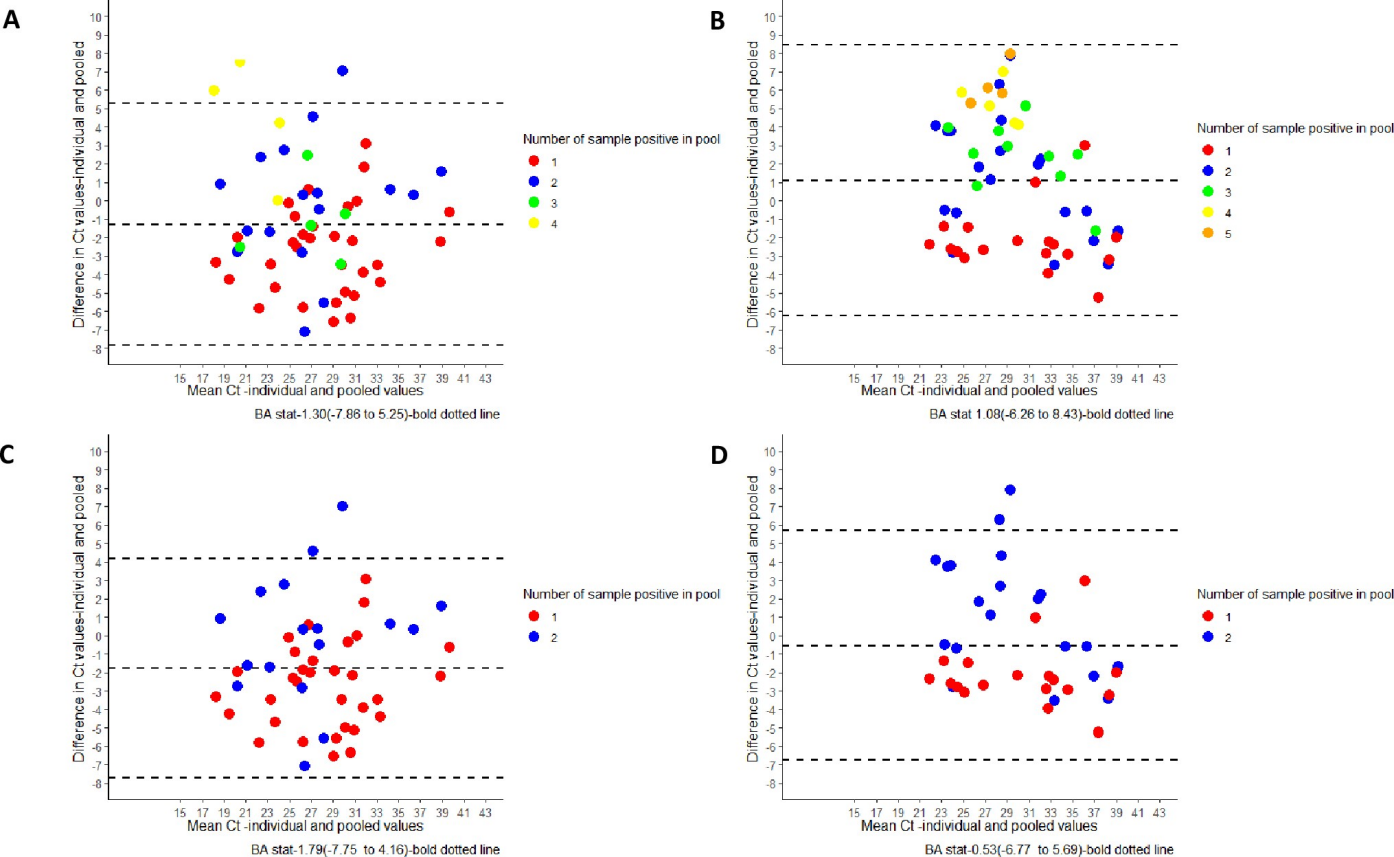
Bland-Altman plot showing the relative positions of Ct values of pooled values in reference with individual Ct and bias estimates between (A) 4-sample pools and (B) 5-sample pools, (C) 4-sample pools with 1 and 2 positives samples and (D) 5-sample pools with 1 and 2 positives samples. Colored dots represent the number of positive samples in a pool.

In order to explore the pattern behind the improved diagnostic sensitivity of the 4-sample pool over 5-sample pool testing we generated BA plots to compare the estimate of bias between mean (individual and pool Ct) values and difference (individual—pool Ct) values. The estimate of bias was -1.30 (-7.86 to 5.25) for 4-sample pools while the same was 1.08 (-6.26 to 8.43) for 5-sample pools, suggesting a greater bias in agreement in 5-sample pools (Fig 1A and 1B). As the issue of discordance was evidently more in 5-sample pools with one positive sample, we further generated sub group BA plots for pools containing 1 and 2 positive samples. This showed a BA stat value as -1.79 (-7.75 to 4.16) for 4-sample pool and -0.53 (-6.77 to 5.69) for 5-sample pools further indicating a greater binary bias (in terms of detection at relatively low viral load and resultant high Ct values) in 5 sample pools (Fig 1C and 1D).

**Fig 2:**
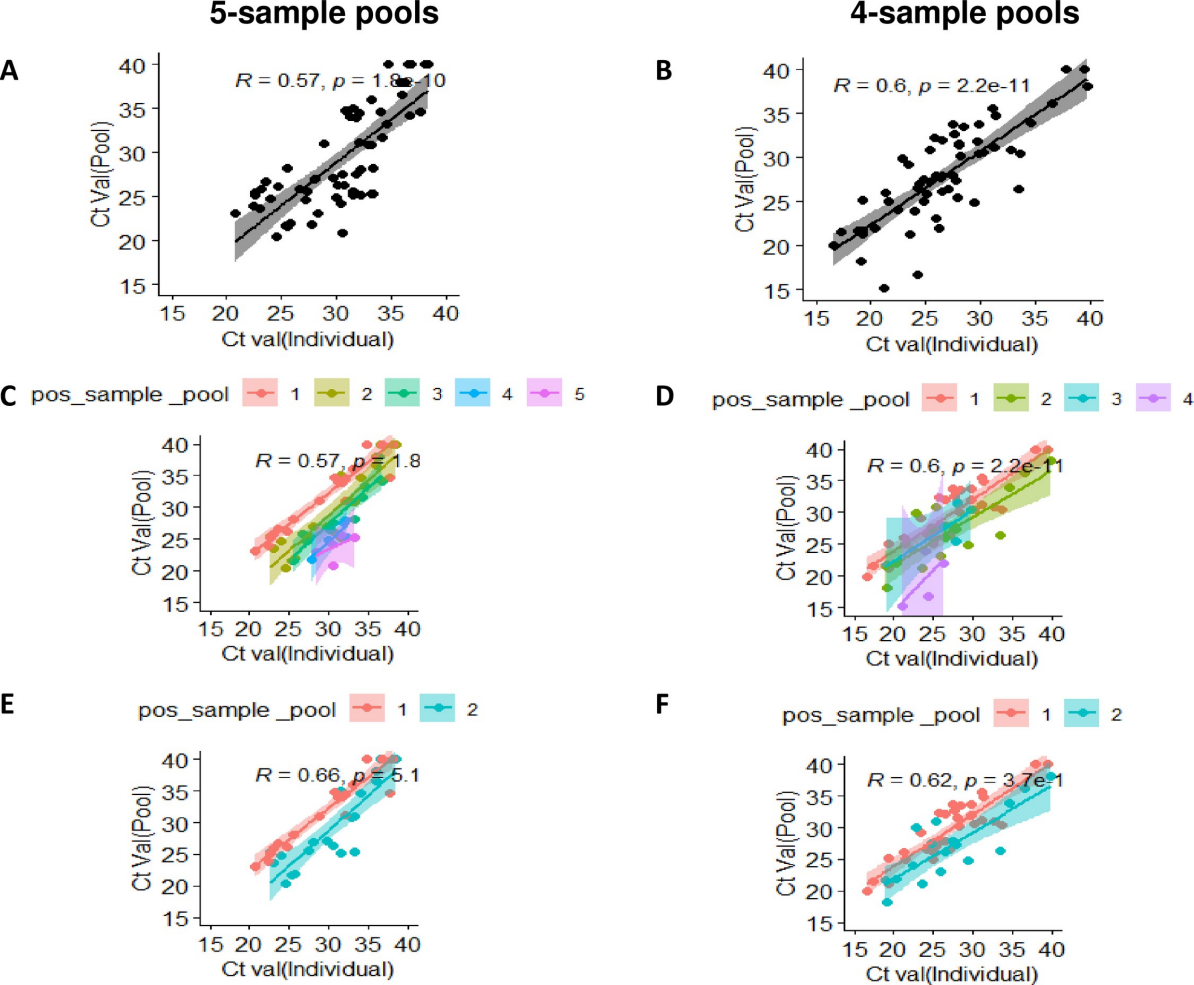
Paired correlation between individual Ct value and corresponding pool Ct value with Kendall tau’s coefficient and slopes. (A-B) 4 and 5-sample pools with all positive samples (C-D) 4 and 5-sample pools with individual slopes for all positive samples in the pool, (E-F) 4 and 5-sample pools with individual slops for 1 and 2 positive samples in the pool.

We further analyzed the extent of correlation between the Ct values of individual rRT-PCR positive samples and their corresponding pools, with and without reference to the number of positive samples in the pools by Kendall’s tau coefficient measurements. Compared to the 5-sample pools, the effect of the number of positive samples on the pool Ct was found to be reduced in the 4-sample pools, thereby suggesting the improved prospect of the latter in providing agreement of results between individualized and pool testing (Fig 2). The comparative diagnostic performance of 4 and 5-sample testing strategies is depicted in Table 3.

**Table 3:** Performance characteristics of the 4 and 5 samples pooling strategy.

Statistic	5-sample pooling strategy		4-sample pooling strategy			
	Simulated Pooling (5 sample pool)		Consecutive pooling: Scenario 1 (Background community prevalence: 2.7%)		Consecutive pooling: Scenario 2 (Background community prevalence: 12.4%)	
	Value	95% CI	Value	95% CI	Value	95% CI
Sensitivity	98.33%	91.06% to 99.96%	100%	69.15% to 100%	100%	92.75% to 100%
Specificity	98.00%	89.35% to 99.95%	100%	96.84% to 100%	100%	95.26% to 100%
Positive Predictive Value	98.33%	89.44% to 99.76%	100%		100%	
Negative Predictive Value	98.00%	87.52% to 99.71%	100%		100%	
Positive Likelihood Ratio	49.17	7.06 to 342.34	100%		100%	
Negative Likelihood Ratio	0.02	0.00 to 0.12	100%		100%	

### Four sample pooling reduces cost and turnaround time in different prevalence settings

While testing 4-sample pools in scenario 1, we found that out of the 125 pools, 10 tested positive. Upon deconvolution of the positive pools the total number of the tests required to diagnose 500 people came down to 171 including the controls. This resulted in a calculated saving of 66% in terms of reagent costs when compared to the individualized testing where a total of 518 tests would have been required including the controls. Similarly, the average turnaround time for testing 500 samples by 4-sample pools decreased by 59%. However, the savings on resources and time was lesser in scenario 2 where the background prevalence was 12.4%. Out of 125 pools, 49 tested positive. Upon deconvolution a total of 333 tests including the controls were required to diagnose 500 samples. Here the calculated savings on resources was found to be 36% and turnaround time was reduced by 30%.

## Discussion

Here we report the absolute concordance between individualized and pooled testing for COVID-19 RT-PCR assay, provided that the pool is restricted to 4 samples. This study, based on both simulated and consecutive pooling, builds on our previous report wherein we have shown that following the strategy of 5-sample pooling leads to a compromised sensitivity, particularly in samples with relatively low viral titer.

By deliberately including a significant number of samples with high Ct values during simulated pooling and performing the analysis across 2 different prevalence settings, our findings demonstrate agreement with reported mathematical models which predict that 4-sample pools are optimal for COVID-19 testing [18, 19]. Since RT-PCR is currently the only recognized molecular modality for COVID-19 testing and considering that this technique involves significant investments in terms of skilled manpower, reagents and processing time, the pooling strategy appears to be an attractive alternative for meeting the increasing demand for testing. Accordingly, several groups, including us, have evaluated the cost and time benefit of pooled testing for COVID-19 diagnosis and found that pooling can substantially reduce the cost and turnaround time depending on the pool size and disease prevalence [11–13]. However, in many of these studies including ours [15], the authors observed a compromised diagnostic sensitivity when compared to the individualized testing [14, 16, 17] leaving behind a pertinent question; what is the perfect pool size for COVID-19 testing?

Performance of pool sample testing is reported to be influenced by pool size, number of positive sample(s) in the pool and Ct value(s) of the positive samples in the pool [14, 15, 20]. We adopted the strategy of simulated pooling with pre-determined proportion of positive samples in the 5-sample pool format as it was otherwise not feasible to ascertain the exact impact of variables like number of positive sample(s) and their Ct value(s) on the diagnostic sensitivity of rRT-PCR assay. In line with our previous studies, such simulated 5-sample pools also demonstrated discordance of 5%. Surmising that over-dilution of the positive sample with high Ct value was leading to false negative results; we assessed the diagnostic performance of 4-sample pool testing in two different prevalence settings in the same geographical area and recorded 100% concordance between pooled and individualized testing. A greater estimate of bias between mean Ct value and difference value observed for 5-sample pools by BA plot (Fig 1) as well as reduced effect of number of positive samples in pool Ct values for 4-sample pools by Kendall’s tau coefficient measurements are in line with our hypothesis.

The strength of this study lies in conclusively demonstrating that a 4-sample pool does not dilute the sample beyond detection limit of the rRT-PCR assays routinely used for COVID-19 diagnosis and offers the optimum balance between diagnostic performance and resource utilization. PCR positivity on pooled analysis is potentially a function of the number of positive samples in the pool and the viral load in individual positive samples. Our previous study and our present findings suggest that the adoption of a 4-sample pooling strategy effectively addresses both these variables and demonstrates the desired diagnostic performance with significant savings of time and resources. Moreover, duplication of similar performance in settings of diverse prevalence, hints at the robustness of this strategy. Using consecutive samples for creation of 4-sample pools, we also demonstrate the applicability of this strategy in the operational settings of a diagnostic laboratory. Although use of different RNA extraction kits and SARS-CoV-2 RT-PCR kits seem like a limitation of this study, concordance between 4-sample pools and individual testing strategies across varied test reagents reinforce usefulness of this strategy in the real-world scenario. Nonetheless, one limitation of this strategy is that an inappropriately collected and/or stored sample in a negative pool will be reported as negative, which may otherwise be rejected and retested to confirm the true status of the sample.

In conclusion, our findings hint at the applicability of adopting 4-sample pooling strategy for improving the testing capacity of COVID-19 diagnostic laboratories, particularly in resource-constrained situations, without compromising on the diagnostic sensitivity of the RT-PCR assays. Nevertheless, it is advisable that laboratories validate the 4-sample pooling strategy against the disease prevalence in the geographical area they are serving, prior to adopting the same for routine diagnostic application.
